# Current Trends in Wick Structure Construction in Loop Heat Pipes Applications: A Review

**DOI:** 10.3390/ma15165765

**Published:** 2022-08-21

**Authors:** Pawel Szymanski, Dariusz Mikielewicz, Sasan Fooladpanjeh

**Affiliations:** 1 Faculty of Mechanical Engineering and Ship Technology, Gdansk University of Technology, 80-233 Gdansk, Poland; 2 Department of Mechanical Engineering, Islamic Azad University, Shahrood 36199-43189, Iran

**Keywords:** wick structures, loop heat pipe, composite wick, thermal conductivity, additive manufacturing

## Abstract

Thermal control systems have been introduced as an important part of electronic devices, enabling thermal management of their electronic components. Loop heat pipe (LHP) is a passive two-phase heat transfer device with significant potential for numerous applications, such as aerospace applications, high-power LEDs, and solar central receivers. Its advantages are high heat transfer capability, low thermal resistance, long-distance heat transfer, and compact structure. The essential role of wick structures on the performance of LHPs has already been highlighted, but no comprehensive review is available that deals with different parameters such as LHP design and wick size, which are largely decisive and effective in achieving a practical level of thermal transmission governed by wick structures. To rely on this necessity, this article summarizes, analyzes, and classifies advancements in the design and fabrication of wick structures. The main conclusion to be drawn after careful monitoring and weighing of the related literature is that LHPs with composites and additively manufactured wicks show a higher heat transfer coefficient than other conventional structures. Indeed, future works should be focused on the design of more structurally efficient wicks, which may allow us to optimize materials and geometrical parameters of wick structure for higher heat transfer through LHPs.

## 1. Introduction

**Figure 1:**
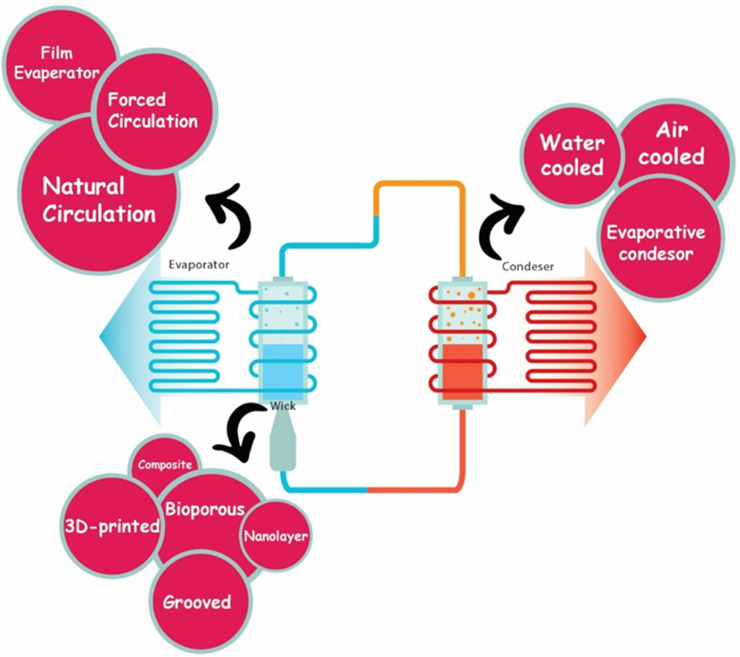
Categorization of different components of an LHP system based on wick structures, evaporators, and condenser.

In recent decades, passive two-phase devices have been developed to solve the thermal control of compact devices with high heat flux and limited space. As highly efficient heat transfer devices, loop heat pipes (LHPs) are highly promising thermal management devices which utilize the phase changing of working fluid to transfer heat and capillary force to drive the fluid cycle [1,2,3]. The history of LHPs originates from 1972, when they were first invented in the former Soviet Union [4]. In comparison with similar two-phase devices such as capillary pumped loops (CPLs) and heat pipes, LHPs have superior heat transfer capabilities, much higher capacity at comparable dimensions, reliable operation in a gravitational field, compact and efficient design, lower thermal resistance, flexible transport lines, minor dependence on the orientation in the gravity field, and considerably higher heat transport capacity for much longer distance [5,6,7,8]. The LHP system consists of different components: an evaporator filled with a fine porous structure (wick), condenser, vapor line, liquid line, and compensation chamber. The components of an LHP are shown in Figure 1, which consists of the main parts and some detailed components.

**Figure 2:**
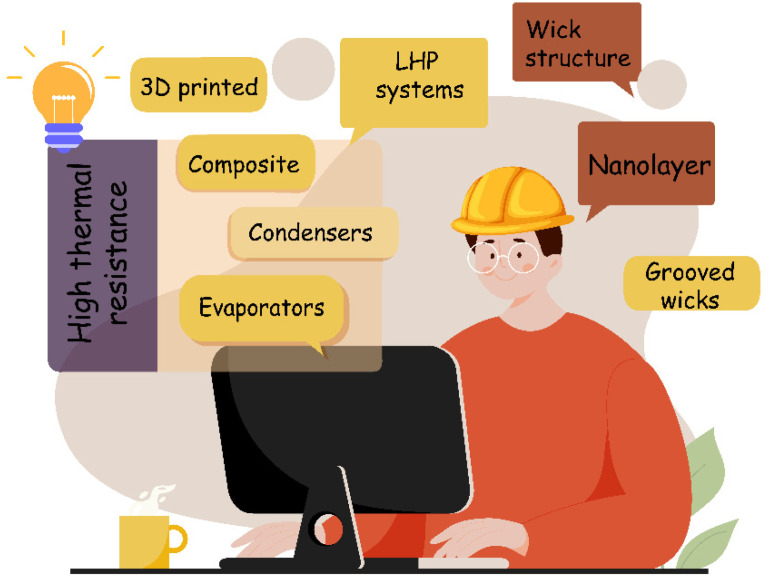
Designs of various LHP components to obtain high efficiency.

Since the beginning of LHP utilization, the performance of this system has been studied numerically and experimentally in many studies [9,10,11,12,13,14,15]. Recently, authors have reviewed the current advances in LHPs with flat evaporators and have discussed various challenges that exist in the LHPs, including uneven distribution of stress in the casing and high internal pressure that causes the evaporator to deform, heat leakage from evaporator heating region and sidewall into the compensation chamber, a start-up with poor performance, reverse flow through the wick, and frequent leaks due to sealing problems [16]. However, the wicks are the most important and at the same time the most difficult component to fabricate in an LHP while fundamentally affecting its performance, and since this subject is a very broad topic, in this article, we want to study the relationship between different wick structures and LHP performance, evaporator efficiency, and thermal resistance, because so far it has not been the subject of any other review article. Various wick structures, such as bi-porous wicks, grooved wicks, nano-layers, composite wicks, and additively manufactured wicks have been investigated and laid in LHPs to provide the necessary capillary force for the automatic circulation of the working fluid in the loop [17,18,19,20,21,22,23]. Moreover, preventing heat leaks from the vapor grooves, from liquid/vapor mutual interference between the vapor grooves, and from the compensation chamber is another critical role of these structures [24,25,26]. As mentioned, various factors contribute to increase the thermal resistance, which Figure 2 shows schematically.

Although the importance of LHPs in a wide range of applications has been recognized, work on the wick structures as an important part of LHPs is still in the early stages of its development. In this review, a particular emphasis has been dedicated to providing a broad overview of the topic of LHPs using wick structures based on very recently released publications. In the first section, we briefly introduce some vocational challenges and difficulties that exist in the development of LHPs. Then, we explain the latest wick families studies that play an effective role in the LHP systems. The heat transfer, permeability, thermal resistance, and heat transfer capacity of LHPs have been discussed by considering the performance of wick structures. Herein, the literature are categorized and fully discussed based on the most current wick structures that have been used for the enhancement of LHP performance, which include bi-porous, grooved, nano-layers, composite, and additively manufactured structures. To delve into deeper layers of the trend of important parameter changes such as heat transfer capability and thermal resistance, the most probable wick structure to show a higher heat transfer coefficient is highlighted. It should be noticed that composite wicks, including composite screens and screen-covered grooves, provide high-capillary pressure and high permeability, but this structure requires complex manufacturing steps [27,28,29]. On the other hand, additive manufactured structures, as an alternative to previous structures, allow much greater freedom in defining the wick geometry and properties [30,31]. Since the most important goal in the performance of LHPs is to achieve higher heat transfer, future research can focus on designing and engineering new wick structures.

## 2. Heat Transfer Using LHP

### 2.1. Application of LHPs

At present, the main area of application of LHPs is space technology. These devices can supplement or replace conventional heat pipes and create favourable conditions for further development of this sphere of technology [32,33,34]. Furthermore, electronics and computers are quite a promising sphere for LHP application. The first actual application of LHPs in electronics is for cooling a unit of powerful transistors as a duplicate system [35]. Moreover, computer technology is a new sphere of LHP application, which has been revealed owing to the appearance of miniature and fairly efficient devices. Furthermore, LHPs are suitable for use in building solar hot water systems, owing to their unique features such as highly effective thermal conductance and flexible design embodiment and installation [36,37,38].

### 2.2. Theory of LHP

One of the most challenging issues in the field of the electronic industry is the thermal management of electronics. Miniaturing electronic systems and increasing performance lead to component development and ultimately lead to increased heat dissipation [39,40,41]. Cooling techniques are the best solution to transfer such high heat fluxes, of which, among the available techniques, two-phase capillary thermal control devices such as heat pipes (HPs), micro heat pipes (MHPs), capillary pumped loops (CPLs), and LHPs are especially promising [42,43,44,45,46]. All these devices are self-circulating, where heat is removed by phase change and the working fluid is circulated by thermodynamic forces. It should be noted that thermal links and fluidics of the compensation chamber to the evaporator have made a fundamental difference between LHPs and other two-phase capillary thermal control devices, and have made a large impact on the design and operation of the capillary loop [47,48,49]. LHPs are referred to as complex systems, in which thermal and hydrodynamic mechanisms between the various LHP components are strongly coupled, thus making it a good replacing and competing technology.

**Figure 3:**
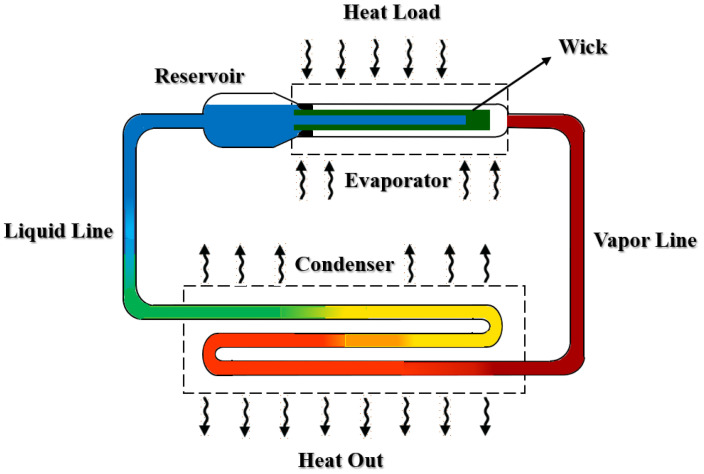
Principal geometry scheme of an LHP in components detail.

An LHP system works on the basis of a simple mechanism with similar physical processes as conventional heat pipes. In a very classical definition, LHPs operate passively by means of capillary pressure generated in the capillary evaporator, which is in thermal contact with the heat source. The load applying the evaporator causes the liquid to evaporate on the outer surface of the wick, which leads to the formation of menisci in the evaporator wick. This causes the vapor collected under the micro-grooves to be transferred to the condenser by the capillary pressure created through the vapor line. This part of the LHP is where the transferred vapors condense by the radiator. The liquid transfer line provides the return path of the condensed liquid transfer to the compensation chamber and the evaporator wick is filled with liquid to complete this circulation (Figure 3). Both the liquid and vapor lines are made of small-diameter tubing that can be easily arranged in tight spaces around the electronic devices. Capillary force is known as the source of fluid circulation in the system which is produced in the evaporator wick and therefore any external power is not required in an LHP performance.

In recent years, extensive research has been undertaken on the performance of LHPs, some of which is listed in Table 1. As mentioned earlier, the performance of LHPs is greatly influenced by different parameters such as wicks and working fluid used in the LHPs, which determines its operating temperature range, heat transport capability and heat transfer performance. Abundant availability in nature and high specific heat has made water a mostly used working fluid in LHPs. As can be seen in Table 1, water is introduced as a working fluid which plays an important role in LHP performance in different ranges of power. The results show that water as a working fluid affects LHP efficiency in different parameters such as the startup, heat transfer coefficient, and thermal resistance. It should be noticed that the flat LHPs are easier to mount on the surface of a hot source without a saddle and have a higher efficiency than other shapes of an LHP i.e., disc, square, and rectangular. Thus, flat LHPs are often preferred for the thermal management of electronic packages for space as well as ground applications [50,51,52,53,54]. Water is not a suitable working fluid in spacecraft as the nonoperating temperature of spacecraft electronics can go down to −40 °C. On the other hand, ammonia has been introduced as a high-pressure fluid that requires a thick-walled container to withstand the high pressures that are unsafe in human space programs. In this regard, acetone and ethanol are less hazardous for the thermal management of small electronics with heat dissipation up to ∼100 W [55,56,57,58]. High-grade acetone is a suitable option as the working fluid of an LHP, where the heat load to be dissipated is fairly small. Acetone presents several advantages such as much reduced handling risks, near atmospheric pressure working pressure in the ambient temperature range, less expensive distillation/purification process and filling devices, and relatively high liquid surface tension. Furthermore, charging an LHP with acetone causes its pressure to approach atmospheric pressure, which reduces the risk of leakage [59]. In order to avoid the occurrence of cavitation and get a better character of the LHPs, ammonia is introduced as a suitable working fluid [60]. However, due to the negative effects of ammonia on health, other working fluids can be replaced to decrease the risk and expand the application area of LHPs. Some other candidate working fluids might be suitable for LHPs. Typical working fluids of this kind include acetone, methane, methanol, and R245fa. Nowadays, new working fluids such as R245fa are also considered for LHP applications [61,62,63]. 

## 3. Wick Structures

**Figure 4:**
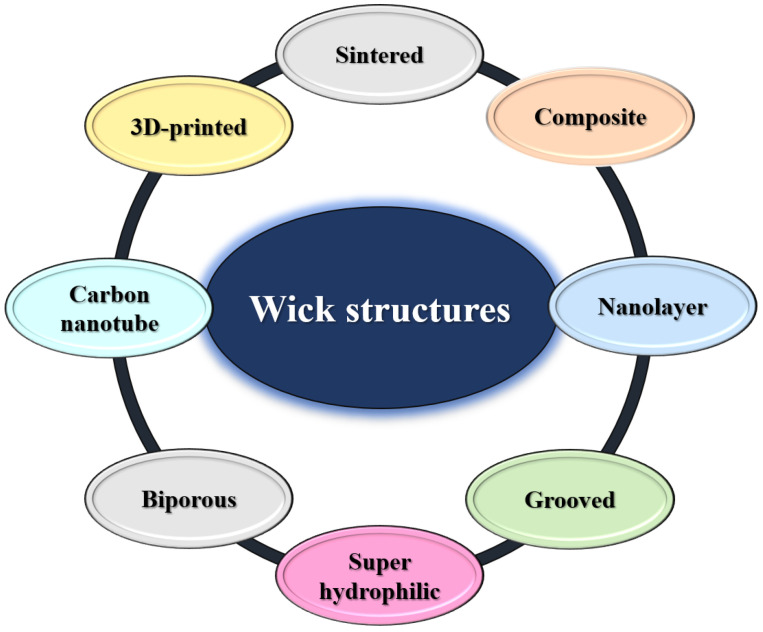
The most common wick types are sintered powder, metal meshes, composite wicks, sintered metal fibers, grooved wicks, additively manufactured wicks, mesh-groove wicks, and bi-porous wicks.

According to the previous section and components of the LHP system, the main source of power for the whole system is provided by the capillary force supplied by the wick and is therefore introduced as one of the most important parts of the LHP system, whose wick structure is mentioned as a key determining factor in LHP performance. For this reason, wick structures are introduced as the most crucial components for fabricating an LHP. The wick can be described as a porous medium with a capillary pore structure. Permeability, effective thermal conductivity, and maximum capillary force are defined as macroscopic properties or performances that are determined by the size, shape of the pores, and porosity [80,81,82]. High permeability to lower the flow resistance of liquid flow, low thermal conductivity to reduce the through-plane heat leakage, and capillary force to prevent vapor from penetrating the wick and entering the liquid line have been introduced as the main requirements of an ideal wick [83]. The most popular class of LHP wicks includes bi-porous, screen mesh, composite, grooved, and additively manufactured wicks. Figure 4 is shown to better express the classification of wick structures.

### 3.1. Review of Thermal Conductivity

Thermal conductivity is a measure of a substance’s ability to transfer heat through a material by conduction, which is an important parameter for LHP systems. In recent decades, extensive research has been done on LHPs to calculate and report thermal conductivity properties. To study the thermal conductivity in an LHP system, Li et al. [84] proposed porous Si_3_N_4_ ceramics with monomodal and bimodal pore size distribution selected as novel wick materials for LHP. Since the capillary and thermal performance of porous Si_3_N_4_ wicks is influenced by tailoring their pore structures, the effects of pore structures including porosity, pore size and tortuosity on the capillary and thermal performance of porous Si_3_N_4_ wicks were investigated. The comparison of experimental values and calculated values of effective thermal conductivity indicates that the most suitable calculated curve is provided by the Alexander model based on limited data in the test. Moreover, the wicks with higher total porosity exhibit lower effective thermal conductivity and the wicks with monomodal pore size distribution show larger effective thermal conductivity compared with those with bi-modal pore size distribution owing to their lower porosity and homogeneous pore size distribution.

Liu et al. [85] used a thin carbon fiber based on polyacrylonitrile to study the working characteristics of a flat-plate LHP. To increase the hydrophilic properties of carbon fiber, they used a chemical plating process in which the surface of the carbon fiber was coated with copper, which turned the carbon fiber surface into a hydrophilic material with an excellent capillary force. Their experimental results indicate that the thermal conductivity of the surface increases with the copper plating layer and the working fluid is an effective element for uniform heating in the evaporation chamber.

**Figure 5:**
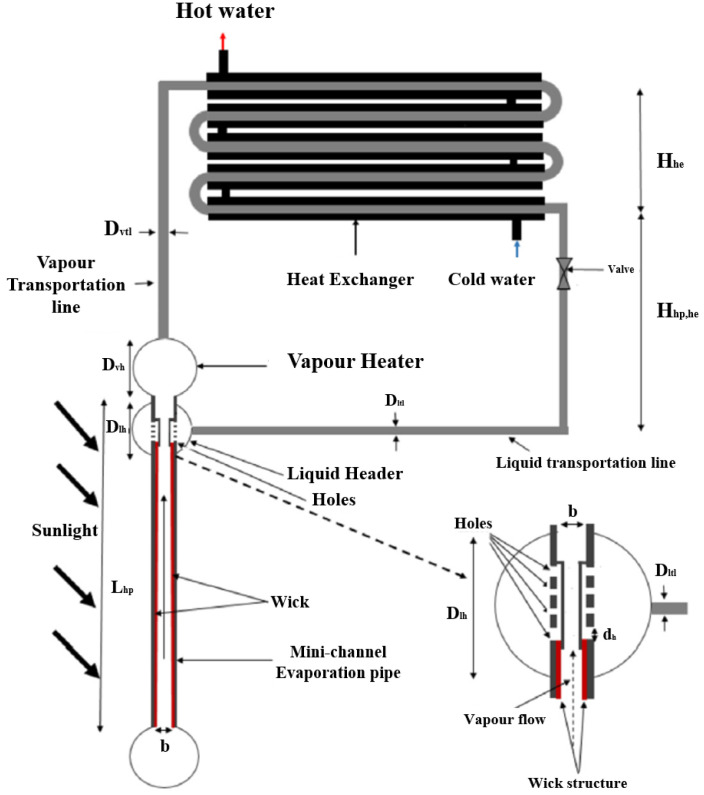
A schematic of a new design for the condensate liquid return path comprising the liquid transportation line and liquid upper feed header [86].

In another study, the impact of the fractal geometrical parameters of the wick on the heat transport capacity of the micro-channel loop heat pipe (MCLHP) was investigated by treating the wick of the microchannel evaporator of the MCLHP as a thin porous layer, which can establish correlations between the thermal parameters of the MCLHP and a number of fractal elements of the wick [86]. Figure 5 shows a schematic of the novel solar LHP, the refrigerant flow, and evaporation in the micro-channel with a porous wick. The authors report that increasing the solid volume fraction was directly related to reducing the effective porosity and pore section, and therefore the higher the boiling point of the MCLHP and the lower the risk of drying out the evaporator’s inner surface. A lower wick effective thermal conductivity is created by reducing the tortuosity fractal dimension and tortuosity of capillaries of the wick, which results in higher thermal resistance in the wick, a reduced boiling point of the MCLHP, and faster dry out of the fluid at a lower heat transfer rate.

### 3.2. Heat Transfer Coefficient

**Figure 6:**
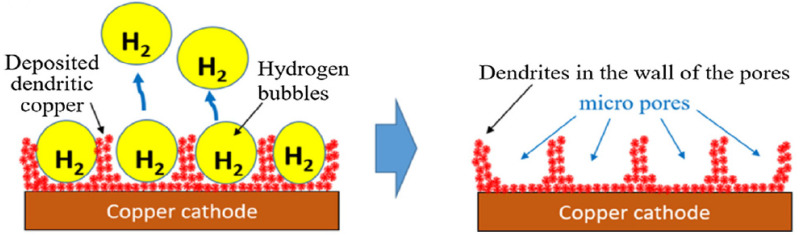
Schematic illustration of a micro-nano bi-porous copper surface with hydrogen bubbles departing the cathode using the dynamic bubble template electrodeposition method [90].

The proportionality constant between the heat flux and the thermodynamic driving force for the flow of heat provides the coefficient of heat transfer in thermodynamics and mechanics. This parameter is mentioned as a quantitative characteristic of convective heat transfer between a fluid and the surface low over by the fluid [87,88,89]. Wang et al. [90] prepared micro-nano bi-porous copper surface structure to investigate the heat transfer coefficient. They utilized the original micro-nano bi-porous copper surface as the first sample and the modified micro-nano bi-porous copper surface as the second sample to compare with conventional surfaces. The first sample was prepared using a hydrogen bubble template deposition method to form abundant pores in optimal cavity size (Figure 6), and the other was modified by applying a low current density to the first sample. The original micro-nano bi-porous copper surface (first sample) was made as cylinders with a diameter of 19 mm and thickness of 8 mm. The thickness of the second sample was higher than that of the first one. Their results show that the increase in heat flux and heat transfer coefficient is directly related and the increase in heat flux increases the heat transfer coefficient. Both of the micro-nano samples had a superior heat transfer coefficient than the plain copper surface at the same heat flux. The main results show that the heat transfer coefficient of the first and second samples were 2.8- and 4.8-times over the plain surface, which are equal to 13 W.cm^−2^.K^−1^ and 23 W.cm^−2^.K^−1^, respectively.

Giraudon et al. [91] experimentally investigated the effect of the characteristics of porous wicks integrated into a flat disk-shaped LHP evaporator on their thermal performance and operating limits. The parameters of porosity, pore radius, thickness, static contact angle and permeability were investigated for several porous structures. Eight samples for the thermal study were selected, including porosity, effective pore radius, thickness, and permeability variance in the ranges of 22.5–41.8%, less than 5 μm–18.3 μm, 2–6 mm, and 0.52 × 10^−14^–16.9 × 10^−14^ m^2^, respectively. Additionally, they used water and pentane as the working fluid. Their results show that the heat transfer coefficient between the evaporator wall and the evaporating fluid is significantly higher with pentane than with water. Furthermore, the heat transfer coefficient reached a maximum value of 2340 Wm^−2^.K^−1^ with water and 5310 Wm^−2^.K^−1^ with pentane, but no clear tendency could be highlighted concerning the effect of the wick characteristics on this parameter.

A cost-effective hybrid wicking structure, that can be manufactured in a scalable way using commercial copper micro-meshes along with simple etching processes, to enable a novel capillary-driven liquid film boiling heat transfer by simultaneously improving liquid supply and increasing nucleation sites was examined by Wen et al. [92]. It should be noted that the interconnected microchannels between woven mesh layers and the substrate serve as low flow resistance passages for a high volumetric flow rate of the liquid. The authors have used different samples with a thickness of 85–370 µm and a porosity of 50.9–57.3% for mesh structures. The experimental results indicate that the largest heat transfer coefficient of 138.7 kW/m^2^K is obtained for two samples of hybrid mesh wicking structures with microcavities of 235 and 370 µm in thickness and 53.7% and 50.9% in porosity. The authors believe that the plural of microcavities on the mesh surface decreases the superheat for the early onset of nucleate boiling, resulting in a higher heat transfer coefficient.

**Figure 7:**
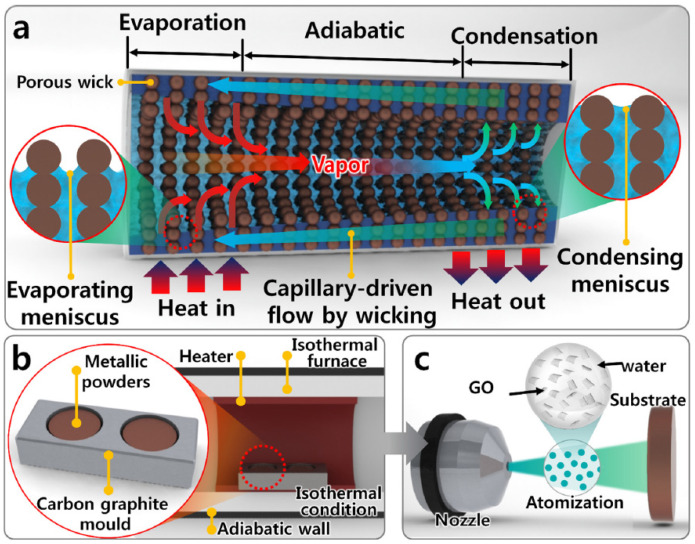
(**a**) Schematic of a heat pipe with a two-phase flow loop, (**b**) sinter of non-uniform copper particles at 600 °C using an isothermal furnace under a vacuum, and (**c**) suspension of graphene oxides in distilled water and the process of spraying this solution on copper wicks [94].

A microporous structure can be provided using copper microparticles, which in and of itself is a wicking medium. Moreover, the wettability parameter increases through the coating of this wicking surface with graphene oxide. Furthermore, the pool boiling performance was examined by mounting the graphene oxide-textured copper wicking surface onto a heated copper plate [93]. The role of the graphene oxide layer is to increase capillary pumping forces. Jo et al. [94] demonstrated the capillary pressure and boiling regime of micro-porous wicks textured with graphene oxide. A porous wicking surface, which contains a micro-porous structure, was designed to promote capillary-driven flow. Using spray deposition, graphene oxides were textured on micro-porous wicks heated by a hot plate with layer thicknesses controlled by the amount of graphene oxide sprayed. The main purpose of the authors was to demonstrate that micro-porous copper coated with graphene oxide has elevated capillary forces that can increase both the critical heat flux and the convective heat transfer coefficient. A two-phase LHP, micro-porous wick, and spray process of graphene oxide onto copper wicks are depicted in Figure 7. Three samples of multilayer, mono-porous, and porous wicks coated with graphene oxide solution were studied to investigate the heat transfer coefficient element. Boiling heat transfer in graphene oxide coatings is higher than mono-porous and uncoated wicks. Due to the hydrophilicity of multi-layered and porous wicks coated with graphene oxide, their bubble production rate is higher than mono-porous wicks. The heat transfer coefficient element increases with increasing number of nucleation sites by the following porous structure.

### 3.3. Capillary Performance

**Figure 8:**
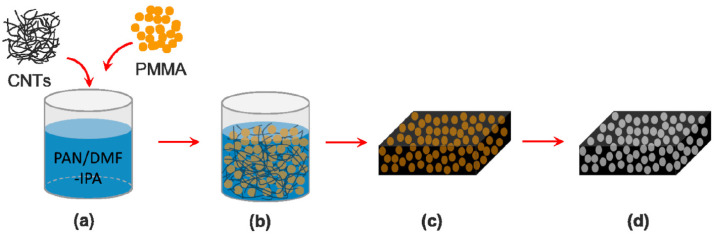
Schematic illustration of the fabrication process for preparing NC foam using the template method and PMMA microspheres. (**a**) The CNTs were dispersed in Polyacrylonitrile (PAN)/Dimethylformamide (DMF)- Isopropyl alcohol (IPA) solution and then Poly methyl methacrylate (PMMA) microspheres were added in. (**b**) The well-dispersed CNT and PMMA microspheres in PAN/DMF-IPA solution after sonication. (**c**) The mixture of CNTs/PAN/PMMA microspheres after filtration. (**d**) The NC foam after thermal treatments [95].

Many researchers have used different wick structures to improve the performance of LHPs. They have studied various elements such as porosity and pore size to investigate the changes in capillary action of the wick. Li and Zhang [95] examined the bi-porous nanocarbon (NC) foams and the effect of the structure with different pore sizes and densities of the samples on capillary performance. The fabrication process is shown in Figure 8. Different pore sizes of 9 to 65 µm and different densities of 26.5 to 144.4 mg/cm^3^ for nanocarbon foams were compared. All samples contained around 75 wt% carbon nanotubes (CNTs) and around 25 wt% graphitic carbon. They found that the capillary performance of the NC foams reduces by more than 60 mg/cm^3^ of density. Moreover, the increase in the micro-pore sizes resulted in an increase in height and velocity of capillary rise. Furthermore, they studied the capillary performance of the foams placed either perpendicular (90°) or parallel (0°) to the surface of the working fluid. The foam at 0° orientation gave a faster and higher capillary rise at the same time compared with that at 90°. Because the NC foams have an isotropic structure, the difference is mainly due to gravity.

Several factors affecting the performance of the wick structures can be expressed: Inverse ratio of capillary force to depth according to the Laplace–Yong equation [96], increase the capillary force by decreasing effective pore diameter and increasing capillary force by decreasing contact angle. Zhang et al. [97] examined an application of bi-porous wick in a flat LHP with a long heat transition distance and introduced an operating mechanism. They used a bi-porous wick to test the performance limit in different conditions. Moreover, a flat-plate LHP was made by clarifying the manufacturing process and the working process. Lack of sufficient subcooled fluid causes the formation of the vapor phase in the compensation chamber, which leads to temperature pulsation in the range of low heat load in horizontal conditions, and the reason for this can be high flow resistance in the long liquid line and the effect of heat leakage. Powder preparation, molding, sintering, and post-processing are the manufacturing processes of a bi-porous wick. The formation of small and large pores by nickel powders and the dissolving of the pores, respectively, reduces the flow resistance in the wick without reducing the capillary force.

**Figure 9:**
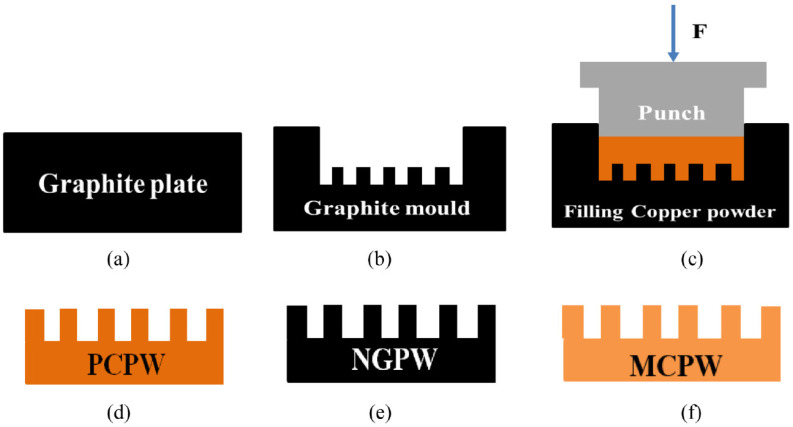
A schematic representation of the sample preparation processes form graphite plate to MCPW. (**a**,**b**) A micro grooved graphite mold is machined on a graphite plate by a computer numerical control machine. (**c**) Solid-phase sintering is carried out by filling the graphite mold with the copper powder. (**d**) PCPWs are finally separated from the graphite mold. (**e**) The cleaned samples which washed in acetone and ethanol solution are put into an oven and dried at 50 ◦C and finally, the etched sintered-powder wicks form a dense layer of oxide nanostructures (NGPWs). (**f**) Nano grass in the sintered powder wick is removed by a chemical cleaning process to leave porous structures covered by high density microcavities (MCPWs) [98].

Capillary performance of LHP systems is a key indicator, which leads to improved LHP efficiency. Two experiments of capillary pumping and forced liquid flow were performed to determine the permeability using porous structures of different sizes by Lee et al. [98]. Using three plain sintered-powder wicks (PCPWs), sintered-powder wicks covered by nano-grass (NGPWs), and the composite wick covered by microcavities (MCPWs) shown in Figure 9, they found that PCPW, NGPW, and MCPW had higher capillary pumping rates and higher wicking velocity in the capillary pumping process. The maximum capillary pumping amount increased by 73%.

Table 2 presents a comparison between recent works related to wick structures with working fluids. Moreover, the value of power and thermal resistance is summarized in this table. It should be noticed that in some cases, if the value of power and thermal resistance were not available, “N/A” was used. Furthermore, different parameters such as thermal conductivity, permeability, and capillary performance of LHP systems are reported. 

It is important to study the role of the wick structures carefully as they are an important part of LHPs. Wick structures affect the thermal properties of LHP systems by pumping the working fluid back to the evaporator surface from the condenser section and transferring heat from the evaporator surface. As mentioned above, a lot of research has been done on the wick’s family and various parameters, such as permeability, heat transfer coefficient, thermal conductivity, and capillary forces, have been compared with each other in terms of how they affect the performance of LHPs. The use of bi-porous, composite, 3D printed, and non-metallic wicks such as silicon, ceramic and polytetrafluoroethylene has resulted in an increase in start-up time which consequently resulted in the decline of heat transfer characteristics due to its low thermal conductivity [129,130,131]. As can be seen from Table 2, equipping LHP systems with porous wick structures can be accompanied by a quick response to thermal loads and good start-up performance. Furthermore, the obtained values of permeability for different samples show that the optimal value of this parameter depends on various elements such as the porosity, temperature and powder size. It is crystal clear that the thermal resistance of the samples with grooved wick structures decreased more than in other wicks. The intensification of the liquid phase change in the evaporator section and the decrease of the liquid level in the condenser section can be considered one of the main reasons. Since the increase in the heat transfer coefficient of the condensation is related to the condensed vapor on the wick, this causes the condensate thermal resistance to decrease rapidly [115]. Furthermore, the reported values for heat transfer coefficient as another parameter affecting the performance of LHPs, show that it can reach the optimum value for wick structures, which is affected by the choice of temperature and particle size [132,133].

Generally, the wick structures, as important component parts of LHPs, produce the necessary capillary force for the automatic circulation of the working fluid in LHP systems. The structural characteristics of the wicks, such as pore radius, permeability, porosity, and effective thermal conductivity, are determined by the properties of internal structure, material, and manufacturing method. Although sintered wicks are the most common technology to fabricate the capillary wick, these structures have some problems, including the drawbacks of random internal structure and low mechanical strength. The wick’s performance is strongly influenced by these structural issues, which limit production. Capillary pressure and its permeability to the working fluid are considered key indicators for wick performance. The most ideal structure would be able to create high permeability and capillary pressure. A new type of wick, the screen mesh wick, could eliminate the lack of a random internal structure; however, the shape of the internal wick passage and the geometric size of the pore could not be controlled. It should be noticed that most of these structures are made of sintered metal powder (copper, stainless steel, and titanium), which has high thermal conductivity; however, this property causes heat leaks during the operation and leads to a high saturation temperature. Recently, much research has been devoted to more advanced composites. Composite wicks provide high-capillary pressure and permeability. Moreover, composite structures reduce the startup time and operating temperature of LHPs and have lower heat leaking which is a good solution to the optimization of heat transfer performance. However, composite wick construction requires complex manufacturing steps. Apart from these traditional wicks, additive manufacturing, colloquially known as 3D printing, provides an alternative method to manufacture capillary wicks. This method can easily control the geometric size and shape of the internal wick passages and eliminate the randomness of the internal structure, unlike conventional manufacturing processes. Additive manufacturing allows for the production of complex wicks with desired parameters such as porosity, permeability and thickness as well as desired porosity where pores are evenly distributed. Wicks that are 3D printed can be produced and integrated by making a freeform porous structure with complex geometry without introducing more interfaces and flexibility. This process will result in a significant improvement in the rate with which heat can be removed, potentially leading to an important breakthrough in thermal management systems. A 3D-printed wick structure could well be an alternative for traditional structures, offering small-scale feature-sized and 3D ligament arrangements in a variety of possible configurations. Moreover, the additive manufacturing method allows LHP to save labor costs and production time [30].

## 4. Conclusions and Future Directions

The performance of electronic devices, on spacecraft and terrestrial applications, is a major challenge for researchers. As a kind of fast heat transfer device, LHP systems, due to their high heat transfer capability, flexible transport lines, long-distance heat transfer, and anti-gravity heat transfer have been widely used in the heat control of equipment. LHPs have different parts, of which the wick structures are the core component. The family of wick components, including bi-porous, nanolayer, composite, sintered, grooved, and 3D-printed wicks, provide a capillary force to circulate the two-phase working fluid and they act as the primary location for a phase change heat transfer. Multiple research groups worldwide have been focused on enhancing and developing the efficiency of wick structures in LHP systems. The current review paper is an attempt to better understand the effect of wick structures on LHP efficiency which leads to realizing different parameters such as thermal conductivity, permeability, thermal resistance, and wettability. In this study, types of wick families were introduced, with 3D-printed wicks as the most beneficial type.

Additive manufacturing technology can directly produce complex 3D parts with a high level of freeform features. This method, as an advanced approach, can offer a range of advantages compared with conventional manufacturing techniques, including unique design, fewer geometric constraints, and a higher production rate. The authors of this review paper believe that recognition of different aspects of 3D-printed wicks could result in the fabrication of heat transfer devices with high performance.

Composite wicks or wicks containing composite additives provide high capillary pumping, high permeability and, as a consequence, improve overall LHP performance. But it should be remembered that composite structures require complex manufacturing steps, and composite wicks after a long period usually react with working fluid and usually cause leakages in LHP installations.

## Figures and Tables

**Table 1:** Comparison between recent works related to the performance of LHPs.

Working Fluid	Power (W)	Property	Effect	Ref.
Water	20–160	LHP through a bypass line	- The bypass line reduced the minimum startup time of the LHP; - The bypass line did not significantly affect the thermal resistance of the LHP.	[64]
Water	20–580	Copper nanowire coating	- Heat transfer coefficient values increased nearly 2.7 times for evaporators with Cu coating; - Thermal resistance values decreased by approximately one-third in nanowire-coated surfaces; - For a maximum heat flux a temperature difference of 8 °C was obtained for all copper nanowire coated surfaces.	[65]
Water	50–300	Oscillating LHP	- LHP could start-up successfully with the working fluid of water.	[66]
Acetone	60–240	Flat evaporator	- LHP transported heat up to 260 W; - The minimum thermal resistance of the LHP was 0.13 K/W.	[67]
Acetone	N/A ^1^	LHP with a nickel wick	- The acetone-charged LHP could successfully realize the startup; - Increase heat transport capability from 60 to 100 W.	[68]
Ethanol	25–180	Miniature LHP with flat evaporator	- There is no bubble generation in the compensation chamber at any heat input prior to deprime.	[69]
Ammonia	6–50	Stainless steel flat LHP	- During the start-up, the superheat needed to start the nucleate boiling is always greater than 10 °C at Q = 6 to 50 W; - The heat transfer capacity is greater than 330 W; - LHP is insensitive to the relative orientation of the evaporator and the CC under gravity.	[70]
Ammonia	2.5–180	Stainless steel LHP with flat disk evaporator	- The heat load is transferred by LHP up to 180 W at a heat sink temperature of −10 °C; - LHP starts up successfully at a minimum heat load of 2.5 W.	[71]
Ammonia	5–370	Stainless steel LHP	- LHP quickly responded to variable heat load and no temperature oscillation occurred; - LHP could handle a maximum heat load of 370 W; - Decrease the thermal resistance of the evaporator by increasing the heat load.	[72]
Ethanol-Water	100–300	Stainless steel-nickel LHP	- LHP with 60% concentration of working fluid mixture showed a lower operating temperature of 178.1 °C; - Mixture of ethanol and water preserve better steady-state heat transfer performance.	[73]
Methane	2–10	Cryogenic LHP	- LHP could realize the supercritical startup successfully with an auxiliary heat load of 2 W; - The methane cryogenic LHP is not sensitive to the charged pressure of the working fluid, and it operates within a relatively large range.	[74]
R245fa	10–160	Evaporator with a strengthened ribbed plate	- Tolerating working pressure for evaporator with the permissible temperature of the normal action for electronics; - The evaporator active zone was greatly extended, effectively reducing the transferred heat flux; - Temperature difference among each monitoring point less than 0.5 °C and chill the heat source evenly.	[75]
R245fa	10–140	LHP with a composite-material evaporator	- LHP showed a good startup performance and a quick response to the heat load recycle at a heat load range of 10 to 140 W; - Maintaining the evaporator heat transfer coefficient at a high level.	[76]
Methanol	N/A	LHP with a bypass line	- LHP has a minimum thermal resistance of 0.18 K.W^−1^ at 220 W; - The maximum heat flux is 39.3 W.cm^−2^, maintaining the heating wall temperature within 85 °C.	[77]
Methanol	2–60	Bi-porous nickel wick	- The miniature LHP can successfully start-up at a heat load of 2 W; - Minimum thermal resistance of the evaporator equal to 0.27 °C/W was achieved at a maximum heat load of 60 W.	[78]
Methanol	30–170	Micro/nano-hybrid structures	- The maximum heat transfer coefficient of 42.17 kW/m^2^.K is achieved; - Increased tolerance of the proposed new heating pipe system to a heat flux of 35.12 W/cm^2^.	[79]

^1^ Not available.

**Table 2:** The comparison between different attempts to increase LHP performance by using wick structures.

Wick	Working Fluid	Power (W)	Thermal Resistance	Main Findings	Refs.
Cement-Pouring porous wick	Methanol	10–80	0.2 (W/K)	- LHP can start up at a heat load range from 5 W to 80 W with the evaporator surface temperature below 100 °C; - Increasing the thermal resistance of the evaporator by increasing the heat load at a heat sink temperature of °C.	[99]
Porous cylindrical wick	Pentane	10–100	N/A	- The vapor is able to flow along the grooves instead of penetrating the wick; - Reduce thermal performance with polishing; - Appropriate modification in wettability of the wick and delay in the occurrence of boiling with gold coating; - Increase the thermal performance of the evaporator with the coating; - The coating reduces the strong effect of the surface topography on the optimum heat flux for a zirconia wick; - The gold coating has a positive influence on the optimum heat load of a zirconia sample with a concave surface.	[100]
Porous copper fiber sintered wicks (PCFSWs)	Water- Ethanol	N/A	N/A	- Capillary pumping amount of PCFSWs with homogeneous porosity first increases and then decreases with increasing porosity; - Much larger capillary pumping amount of 90–70% gradient porosity PCFSWs is observed with the high porosity; - Centrifugal tests showed the rough PCFSWs with deionized water and low porosity possess a smaller centrifugal amount; - Ethanol has a larger centrifugal ratio compared with that of deionized water.	[101]
Porous NiO wick	N/A	20–160	N/A	- Heat location, provided by the NiO wick, helped improve the thermal conversion efficiency; - Localized vaporization in the micro channels of the NiO wick’s surface further improves the heat utilization efficiency; - Special macroscopic thermal insulation of NiO wick provided a thermal barrier to minimize heat loss to the brine; - The corresponding heat-conversion efficiency is 65.2% and 90.7%, respectively.	[102]
Microstructure of porous copper wick	N/A	N/A	N/A	- Maximum amount of capillary height was reached 27.5 mm; - Increasing the porosity of the coatings produced an even greater increase in the mass of liquid rising in the copper strips; - The permeability of coatings increases with their effective porosity; - Capillary pressure increases for a given groove size.	[103]
Sintered copper powder wick	Water	10–100	0.05 (°C/W)	- The sample with 75–100 µm performed best in both the thermal resistance and the heat transport capability; - The sample with 75–100 µm could start-up successfully within 10 min without temperature overshoot and oscillation; - The sample with 75–100 µm performed better for its lower operating temperature in gravity-assisted orientation; - The minimum total thermal resistances for the sample with 50–75, 75–100, and 100–125 µm in gravity-assisted orientation are approximately 0.038 °C/W (50 W), 0.042 °C/W (80 W) and 0.049 °C/W (80 W), respectively.	[104]
Sintered porous wicks using stainless steel	Ethanol	N/A	N/A	- The droplets on the spherical powder samples always spread more quickly than those on the irregular powder samples with the same powder sizes; - Permeability of irregular powder samples is much larger than that of the spherical ones with the same powder size range; - The wicks with medium powder size of 90–120 µm, maintained a good balance between permeability and capillary pressure; - For both spherical and irregular powders, the slopes of the pressure drop decrease significantly with the increase of the powder sizes.	[105]
Bi-porous Composite wick-Wick consists of 3 layers	N/A	N/A	N/A	- The sample with the lowest percentage of mass of copper 700 °C has the lowest amount of thermal diffusivity; - Highest evaporation rate and lowest thermal diffusivity of sample B with porosity of 44.7%; - Samples sintered at 700 °C and 750 °C have higher porosity than that sintered at 800 °C; - Optimum value of permeability was obtained for sample B equal to 1.305 × 10^−12^ m^2^; - Evaporation rate is highest for the sample of composition (%Cu) at 60:30:15.	[106]
Biporous structure of multi-walled carbon nanotube (MWCNT)- polyethyleneimine shells	Water	20–100	0.5 (°C/W)	- Thermal resistance was reduced by 14.3% in the 10-bilayer layer-by-layer-assembled MWCNT-PEI coating; - Thicker nanoporous layers did not improve thermal performance; - 40 bilayers reduced volume of microporous structures and increased thermal resistance; - The hydrophilic nature originating from the PEI shells surrounding the MWCNTs chemically contributed to the enhanced interfacial area between the working fluid and heated surfaces; - The nanoporous structures improved thermal energy transport inside the heat pipes; - The layer-by-layer-assembled MWCNT-PEI coating on the copper-sintered microstructure structurally induced enhanced capillary wicking; - 40 bilayers did not exhibit improved thermal performance.	[107]
Micro-grooved wick	Acetone	N/A	N/A	- Thermal resistance remained stable from 0.055 to 0.074 K/W; - The micro-grooved wick with reentrant cavity array vapor chamber showed good start-up performance; - The micro-grooved wick with reentrant cavity array vapor chamber showed competitive thermal performance; - The micro-grooved wick with reentrant cavity array vapor chamber yielded a fast temperature response and low start-up heat load.	[108]
Bi porous spiral woven mesh wick	Water	10–18	0.13 (°C/W)	- FO, FI, and SI UTHPs had the highest thermal resistance and heat transfer capacities with optimal filing ratio; - Reduce thermal resistance of the FI UTHP by 6.32 to 25.9%; - Increasing the filling ratio at the same heating power as before caused the temperature of each point on the UTPHs to decrease; - Increasing the capillary force of the wick improved the unbalanced temperature distribution of UTHP under high power; - The FI UTHP had the lowest evaporation thermal resistance; - SI sample of a UTHP had the lowest condensation thermal resistance; - Increase the thermal resistance of evaporation with increasing heating power.	[109]
Spiral coil wick	Ammonia	40–120	0.05 (K/W)	- At higher heat loads, the axial-grooved heat pipe could maintain the k_efc_ level, but both arterial heat pipes (0.4 and 0.5 mm) slowly dropped in k_efc_; - Coil wick with wire of 0.5 mm diameter achieved a higher value than the wire of 0.4 mm diameter for all heating power for evaporator film coefficients; - Wire of 0.4 mm diameter performed better than the 0.5 mm except at low heat load; - As heat load increased, the thermal resistance sharply rose for the charged container without coil but only grew gently for the arterial heat pipes.	[110]
Gradedmini-grooves Wick	Methyl alcohol	N/A	N/A	- The effective thermal conductivity coefficient of Case 2 (11.666 W.m^−1^K^−1^) is higher than those of Case 1 (10.378 W.m^−1^K^−1^) and Case 3 (9794 W.m^−1^K^−1^); - The capillary radiuses of Case 2 and Case 3 are lower than that of Case 1 at the evaporation section; - The capillary pressure differences of the three cases between the evaporation and condensation sections are 40.20, 42.83, and 46.31 Pa, respectively; - The liquid velocities of Case 2 and Case 3 are higher than that of Case 1; - The vapor velocity of Case 2 is relatively lower than those of Case 1 and Case 3	[111]
A parallel-groove wick, a sintered mesh-groove wick, and a sintered double-layer 200 mesh wick	Water	12–70	N/A	- For the horizontal operation of the composite wick, the Q_max_ of about 60 W is much higher than 21–25 W for the 2 × 200 mesh wick and about 10 W for the groove wick; - Under a tilt angle of 30–90°, the Q_max_ may reach 39–49 W, in contrast, the 2 × 200 mesh wick cannot operate at Q = 14 W under α ≥ 30°.	[112]
Multi-layer wick	Water	5–80	0.18 (°C/W)	- Multi-layer wick heat pipe exhibits a higher heat transfer capacity in the anti-gravity direction; - Critical heat load of the multilayer wick heat pipe was 79 W at a fully anti-gravity orientation.	[113]
Mesh-type wick structure with nanostructured super hydrophilic surface	N/A	0.5–6	N/A	- Nanostructured super hydrophilic surface increases thermal performance compared with that with the conventional base copper surface; - Capillary performance of the single mesh wick is shown to decrease as the wire diameter or pore size increases.	[114]
Hybrid spiral woven mesh (HSWM) wick	Water	10–20	0.02 (°C/W)	- Increasing the distance from the UTHP tail end reduces the temperature of each testing point of the UTHP samples; - Maximum heat transport capacity of the SB and SF UTHPs increased by 33.33–53.85%; - 27.53% to 42.92% reduction for total thermal resistance; - The lowest R_t_ for the SC UTHP with the heating power below 13 W; - Maximum heat transport capacity of the SA and SH UTHP samples were 15 W and 13 W, respectively.	[115]
Single-layer wicks (SW) and composite wicks	Water	40–140	0.22 (°C/W)	- T_ew_ of SW3 and SW4 samples were slightly higher than those of SW1 and SW2 samples at low heat loads; - h_e_ = 24,932 W/m^2^K with highest amount at Q = 120 W in SW3 sample; - R_e_ = 0.0568 °C/W and R_LHP_ = 0.186 °C/W with lowest amount in SW3 sample; - LHPs can start-up at 120 s with the composite wicks; - Lower T_ew_ of CW1 and CW2 samples compared to SW3 samples through out the operation, and proximity of the heat transfer coefficients of CW1, CW2, and SW3 at the heat loads from 40 to 80 W; - The optimal particle size ranges for CW2 were 48–96 μm for the evaporation layer and 96–180 μm for the transportation layer.	[116]
Ceramic, steel-nickel, and copper wick	Ammonia	10–60	N/A	- Ceramic wick, with the smallest thermal conductivity, showed the lowest heating surface temperature; - Ceramic wick had the shortest start-up time against steel-nickel and cooper wick.	[117]
Plain surface wick, Monolayer wick of the copper sintered particles, Columnar posts wick, Mushroom cap wick	*n*-pentane	N/A	N/A	- The average measured CHF was q_CHF_ = 29 ± 1.5 W/cm^2^ for plain surface; - The average measured CHF was q_CHF_ = 34.8 ± 1.8 W/cm^2^ for monolayer wick which is 20% higher than that of the plain surface; - The average measured CHF was q_CHF_ = 47.8 ± 2.5 W/cm^2^ for columnar posts wick which is 65% higher than that of the plain surface; - The average measured CHF was q_CHF_ = 54.4 ± 2.8 W/cm^2^ for mushroom posts wick which is 87% and 14% higher than that of the plain surface and columnar, respectively.	[118]
Composite wick-Single and two-layer, wick sintered copper particle	Water	20–600	0.052 (K/W)	- Increasing the particle size led to an increase in the dry out; - Two-layer wick shows a near-linear increase in wick superheat as the heat flux increases to 130 W/cm^2^; - Sample C provided the best combination of high dry out heat flux and a low boiling resistance; - Maximum heat flux dissipation of 485 W/cm^2^ over a 1 cm^2^ while also maintaining a low thermal resistance of only 0.052 K.	[119]
The composite wick of sintered copper powder-mesh	Water	N/A	0.1 (K/W)	- The different evaporator and condenser wick structures helped to simultaneously enhance the evaporation and condensation of the working fluid; - Thermal resistance of the vapor chamber decreased by more than 37.67%.	[120]
Composite wick-Single and two-layer wick	Water	N/A	0.1 (K/W)	- Although both these two-layer wick designs had low resistance throughout the boiling curve, the single-layer wick was able to dry out to low heat fluxes; - 400% increase in dry out heat flux for two-layer wick compared to single-layer wick; - The 5 × 5 design has a dry out heat flux of 151 W/cm^2^ at 0.095 K/W (3.4 times larger than a single-layer wick; - The dry out heat flux of the 10 × 10 design was 198 W/cm^2^ at 0.105 K/W, a 4.4 times enhancement over the baseline.	[121]
Composite porous wick with spherical-dendritic powders	Water	10–190	N/A	- The maximum heat load in the composite porous wick from No. 1 to No. 4 is 190 W, 70 W, 110 W, and 90 W, respectively; - The composite porous wick with spherical-dendritic copper powders has the largest critical heat flux of 15.1 W/cm^2^; - Increasing the superheat of the evaporator wall by increasing the heat load up to 40 W; - Startup time decreased with heat load; - The maximum evaporation heat transfer coefficient is 9134.7 W/(m^2^K) for No. 4 sample.	[122]
Multi-scale composite porous wick	Ethanol	N/A	N/A	- Improving the modification of copper powders by MCPW increases capillary performance; - Powder size and type of powder as the morphologies of copper powders affect capillary function; - Nanostructures on the powder surface caused the higher capillary height and rising velocity of working fluid for the wick; - The results of capillary height rise fast for acetone compared with the results of water; - The capillary performance parameter for irregular MCPW is 27.2% higher than that of the spherical-based sample.	[123]
Composite wick-Nickel powder with a size of 2.2–2.8 μm, Copper powder with an average particle size of 13 μm	Water	N/A	N/A	- LHP with a composite wick is found to have a shorter start-up time and lower operation temperature; - Higher capillary pressure during the operation of LHPs with proposed composite wick; - Less heat leakage of composite wick than sintered pure nickel wick; - Effective thermal conductivity is reduced with the addition of copper to the Ni-Cu mixture; - Sintered Ni-Cu wicks have a higher ETC in the wet mode compared to the dry mode.	[124]
Multi-scale composite porous wick	Acetone, ethanol, and water	N/A	N/A	- Provide hydrophilic performance and facilitate the penetration of working fluid into the pores by MCPW; - Increased capillary performance was sintered with irregular copper powder and delayed by NaOH solution; - Both NSP-1 and NSP-2 show an upward trend, while the height of NSP-1 is lower than that of NSP-2 under the same condition; - The prepared NIP samples illustrate larger capillary height than the sintered wicks with irregular powders; - NIP-2 performs better than the H_2_SO_4_ NIP-1.	[125]
Striped super-hydrophilic wick	Water	2–8	0.3 (K/W)	- Minimum thermal resistance was 0.26 K/W with a maximum temperature of 74.07 ◦C at an 8 W heating load; - The low thermal resistance and the maximum temperature for the UTFHP resulted in a filling ratio of 57%.	[126]
3D printed stainless steel wick	Water	20–160	0.2 (K/W)	- 3D-printed wick with high porosity, suitable pore radius, high permeability and low effective thermal conductivity; - LHP could start-up and run successfully at a low heat load of 20 W in about 100 s; - The highest evaporator wall temperature of the LHP with sample A medium and high heat loads; - Temperature oscillations and increase evaporator wall temperature with sample C; - The quality of vapor in the LHP with samples A and B was higher than in sample C; - Thermal resistance increased at a heat load 60 W in sample A; - Increasing the evaporator thermal resistance of sample C due to vapor permeating through the wick; - The highest value of the heat transfer coefficient was up to 44.379 W/m^2^K at a high heat load of 140 W in sample B.	[127]
3D-printed stainless steel porous structure	Water	N/A	N/A	- Increase heat transfer via 3D-printed heat pipe; - The important role of the gravitational effect in the wick structure; - Enhancement of capillary performance through 3D-printed wick.	[128]

## Data Availability

The data supporting the findings of this study is available within the article.
